# Complement Receptor Type 1 (CR1, CD35), the Inhibitor of BCR-Mediated Human B Cell Activation, Differentially Regulates TLR7, and TLR9 Induced Responses

**DOI:** 10.3389/fimmu.2019.01493

**Published:** 2019-07-02

**Authors:** Bernadett Mácsik-Valent, Katinka Nagy, László Fazekas, Anna Erdei

**Affiliations:** ^1^MTA-ELTE Immunology Research Group, Eötvös Loránd University, Budapest, Hungary; ^2^Department of Immunology, Eötvös Loránd University, Budapest, Hungary

**Keywords:** human B cell biology, TLR7/9, complement receptor type 1 (CD35), B cell receptor, activation, proliferation, cytokine- & antibody production

## Abstract

The complement system and Toll-like receptors (TLRs) are essential contributors of innate immunity. Separate activation of these systems has been shown to play a role in initiating and shaping the adaptive immune response, however the modulation of various B cell functions by the simultaneous involvement of these two systems has not yet been uncovered. We demonstrate here that occupancy of complement receptor type 1 (CR1, CD35) by its natural, complement component C3-derived ligand significantly and dose dependently reduces the TLR9-induced expression of activation markers, cytokine production, proliferation, and antibody production by human B cells, but has no effect on the TLR7-induced functions. The synergistic response to the simultaneous engagement of either TLR9 or TLR7 along with the BCR however, is significantly inhibited by CR1 occupancy. Our findings imply that both under physiological and pathological conditions, when complement- and TLR-activating microbial and damage products are present in the B cell environment, the cooperation between CR1 and TLR7 or TLR9 provides additional levels of the regulation of human B cell functions.

## Introduction

The complement system and Toll-like receptors (TLRs) are two major elements of innate immunity, which are critical not only for first line host defense, but also in shaping adaptive immunity. Several microbial or damage products, such as zymosan, LPS, and nucleic acids trigger the complement cascade and initiate TLR-signaling simultaneously, immediately upon encounter with such substances. The developing crosstalk between the two key innate elements may profoundly modulate adaptive immune responses.

B cell functions triggered by the clonally-rearranged antigen-specific B cell receptor (BCR) are known to be regulated by several germ-line encoded receptors—including TLRs and complement receptors (CRs). Simultaneous ligation of TLRs and CRs in conjunction with BCR engagement can occur both under physiological and pathological conditions, when complement-activating and TLR-binding pathogen associated molecular patterns (PAMPs) and danger associated molecular patterns (DAMPs) are present in the B cell environment. Since complement components—in addition to their presence in circulation—can be locally produced and activated in various tissues, C3-derived ligands are easily available for cellular interactions.

TLRs, CRs, and the BCR are all able to bind complexed antigens, thus their interplay may fundamentally alter and fine tune B cell functions. Still, their crosstalk so far had mainly been studied in pairs as separate entities,—such as TLR—BCR or CR—BCR—, not as cooperating players of a more complex system.

The complement system is a basic element of innate immunity, which provides an immediate reaction against invading pathogens. Besides killing them and generating inflammatory response that help fight against infections, it also functions as a bridge between innate and acquired immunity by initiating and regulating adaptive immune responses. The cascade of cleavage reactions generates split products of C3, the central component—including C3b, iC3b, and C3d fragments. A part of these molecules remains covalently attached to the activating surface, providing ligands for complement receptors type 1 (CR1, CD35) and type 2 (CR2, CD21) expressed constitutively on B cells [reviewed in: (1)]. In contrast to mice however, where these receptors are encoded by the same gene and both receptors have been demonstrated to enhance the antibody response (2), in humans CR1 and CR2 are encoded by two different genes, and their function has been demonstrated to be markedly different. Namely, ligation of CR1 has been shown to exert a strong inhibition on various BCR-induced functions—including proliferation and antibody production—both under physiological and pathological conditions (3–7).

TLRs are germ-line encoded transmembrane proteins with an extracellular leucine-rich domain and a conserved, IL-1 receptor homologue cytoplasmic domain (8). They recognize molecular patterns associated with microbial pathogens or damaged self. To date, 10 members of the TLR family (TLR1-10) have been identified in humans (8, 9). Among them, TLR1/2—a heterodimer formed by TLR1 and TLR2, furthermore TLR7, TLR9, and TLR10 have been reported to be expressed both by human tonsil and blood B cells (10). These receptors are activated by bacterial lipoproteins and lipoteichoic acids (TLR1/2), by single-stranded RNA (TLR7) or by un-methylated CpG motifs derived from microbial DNA (TLR9) (8). No ligand is currently known for TLR10 and its role so far seems to be uniqe amongst the known TLRs (11). TLRs are differentially distributed within the cells. In humans, TLR1/2 are expressed on the cell surface, while TLR7 has been shown to be expressed in endosomes (8). Regarding TLR9, both intracellular and cell surface localization have been detected (8, 12–14). Stimulation of human B cells via TLRs with known ligands is known to lead to increased cell survival and proliferation (15, 16), enhanced antigen presentation and antigen specific T cell responses by the up-regulation of MHCII, as well as the co-stimulatory molecules CD69, CD40, and CD80 (10, 15) and secretion of several types of cytokines and chemokines including IL-1, IL-6, TNF-α IL-10, IL-8, MIP-1, and MCP-1 (17). Moreover, TLR engagement induces B cell differentiation into immunoglobulin producing plasma cells (15, 18, 19).

Up to now, the immune response modulating capacity of the complement system and TLRs mainly have been studied separately, and the outcome of their interplay in regulating B cell functions has only scarcely been investigated (20, 21). Therefore, to obtain a deeper insight into the cooperation between these two innate systems, the objective of this study is to reveal how engagement of the inhibitory CR1 influences the activation of human B cells triggered by TLR7 and TLR9, the two most studied sensors of pathogen-derived patterns and damaged self-nucleic acids. Furthermore, we also aim to uncover the functional consequences of the simultaneous ligation of TLR7 or TLR9 and CR1 in conjunction with BCR engagement, which mimics the physiological and pathological conditions, when both complement and TLR-activating microbial or damage products are present in the B cell environment.

We demonstrate here that CR1 clustering significantly and dose dependently reduces the TLR9-induced expression of activation markers, cytokine production, proliferation, and antibody production by tonsillar B cells, but has no effect on the TLR7-induced functions. Importantly however, the synergistic response to the simultaneous engagement of either TLR9 and BCR or TLR7 and BCR was significantly inhibited by CR1 occupancy. Our findings imply that the cooperation between CR1 and TLR7 or TLR9 provides additional levels of the regulation of human B cell functions, which might be important both under physiological and pathological conditions.

## Materials and Methods

### Cell Preparation

Tonsils from children undergoing routine tonsillectomy were obtained from the Saint Istvan and Saint Laszlo Hospital in Budapest. This study was carried out in accordance with the Helsinki Declaration and was approved by the Ethics Committee of the Medical Research Council in Hungary (TUKEB), 52088/2015/EKU. Tonsillar mononuclear cells were isolated by Ficoll-Hypaque gradient centrifugation (GE Healthcare, Chicago, IL. USA). After rosetting with 2-aminoethylisothiouronium bromide-treated (Sigma-Aldrich, St. Louis, MO, USA) sheep erythrocytes, B cells were isolated by centrifugation over Ficoll-Hypaque solution. Separated B cells were further fractionated into low- and high-density populations on a Percoll (Sigma-Aldrich, St. Louis, MO, USA) gradient. All experiments were performed with the high-density (“resting”) tonsillar B cell population. B cell purity was higher than 95% in each case, as verifyed by CD19 expression.

### Isolation of Human C3, Generation of C3b-Like C3

C3 was isolated from pooled normal human serum by fast protein liquid chromatography as described by Basta and Hammer (22). Traces of IgG contamination were eliminated from the C3 solution employing protein G beads (Thermo Scientific, Rockford, IL, USA). The purity of C3 was assessed by SDS-PAGE and ELISA. C3 fractions were stored at −80°C until use. C3b-like C3, the multimeric ligand of CR1 was produced by incubating isolated C3 at 63°C for 20 min. The C3b-like activity of heat aggregated C3 was proven by ELISA and FACS analysis (3), excluding the possibility of its binding to CR2 expressed by B cells.

### Culture Conditions

High-density, “resting” tonsillar B cells were cultured in the presence of different stimuli in RPMI-1640 medium (Sigma-Aldrich, St. Louis, MO, USA) containing 10% FCS (Thermo Scientific, Rockford, IL, USA) and 50 μg/ml gentamycin (Sigma-Aldrich, St. Louis, MO, USA) at 37°C and 5% CO_2_. For assessing the marker expression, cytokine production, and proliferative capacity of the cells 96-well flat bottom culture plates (Corning, Kennebunk, ME, USA), while for the ELISPOT assay round bottom plates were used (Corning, Kennebunk, ME, USA). The suboptimal concentration of the activators was determined in preliminary experiments. Human B cells were activated via BCR using 2 or 5 μg/ml F(ab')_2_ anti-human IgG/M/A (Jackson ImmunoResearch, Cambridgeshire, UK), for stimulation via TLR7 5 μg/ml R-837 (Imiquimod, Invivogen, San Diego, CA, USA) was employed and TLR9 was triggered by 0,5 μg/ml CpG ODN 2006 (Sigma-Aldrich, St. Louis, MO, USA). For CR1 ligation the multimeric natural ligand, C3b-like C3 was used in different concentrations (40, 80, and 120 μg/ml). The ligands were applied either separately or in combinations, as shown in the figures.

### Expression of B Cell Activation Markers

Activation of B cells was monitored through changes in the expression of CD69 and CD40 molecules by flow cytometry, after culturing 2 × 10^5^ cells/well in 100 μl culture medium with the activators for 48 h. Thereafter cells were incubated with FITC-conjugated anti-CD69 (BD Biosciences, San Jose, CA, USA) and APC-conjugated CD40 (Beckman Coulter, Irving, TX, USA) antibodies for 30 min. Staining was assessed by FACSCalibur flow cytometer and the CellQuest software (BD Biosciences) San Jose, CA, USA.

### Cytokine Production

B cells were seeded onto 96-well cell culture plates at the density of 2 × 10^5^ cells/well in 100 μl culture medium, followed by stimulation, as described. After 48 h the IL-6 content of the supernatant was measured using IL-6 Duoset ELISA systems (R&D Systems, Minneapolis, MN, USA), according to the manufacturer's instructions.

### Proliferation Assay

B cells were plated at 4 × 10^5^ cells/well in 100 μl culture medium containing different combinations of activators, as shown in the figures. After 48 h cells were pulsed with 1 μCi/well [^3^H]-thymidine (NEN, Boston, MA, USA) for 18 h. Incorporated radioactivity was measured with a Wallac 1409 liquid scintillation beta counter (Wallac, Allerod, Denmark).

### Antibody Production

To induce immunoglobulin production IL-2, IL-6, and IL-10 (50 ng/ml, each, Immunotools, Friesoythe, Germany) were added to the cells in the presence of different combination of the activators and the CR1 ligand for 3 days at 37°C and 5% CO_2_, as shown in the figures. The frequency of IgM secreting cells was evaluated by enzyme-linked immunosorbent spot (ELISpot) assay according to the manufacturer's guide (MabTech, Nacka Strand, Sweden). Briefly, 96-well Multiscreen-IP Filter Plates (MAIPSWU10, Millipore, Burlington, MA, USA) were coated with 15 μg/ml mouse anti-human IgM (MabTech, Nacka Strand, Sweden) at 4°C, overnight. After washing, 1 × 10^4^ differently treated cells were added to the wells, and incubated at 37°C. After 20 h cells were aspirated and plates were washed and incubated with 1 μg/ml biotinilated mouse anti-human IgM (Mabtech, Nacka Strand, Sweden) detection antibody, followed by horse raddish peroxidase conjugated streptavidin (MabTech, Nacka Strand, Sweden). Spots were developed with 3,3′,5,5′-tetramethylbenzidine (TMB, MabTech, Nacka Strand, Sweden), and counted using the CTL Immunospot Reader (Cellular Technologies, Shaker Heights, OH, USA).

### Statistical Analysis

The relative mean effect of CR1 ligation on different functions was calculated by dividing the experimental data obtained for the activated and C3b-like C3 treated samples with data of samples, where the C3-derived ligand was absent. Results of the activated samples are taken as 100%, and data of CR1 ligand-treated samples are expressed as percentages of the activated samples. Statistical differences were assessed by one-way ANOVA using GraphPad Prism version 6.00 for Windows (GraphPad Software, San Diego, California). Figures were prepared with the same GraphPad Prism software. *P*-values of < 0.05 were considered significant.

## Results

### Effect of CR1 Ligation on Activation Induced Upregulation of CD40 and CD69 on Human B Cells

Upon TLR activation, B cells upregulate costimulatory molecules and enhance antigen presentation to T cells (10, 15, 23). Therefore, in the first set of our experiments we assessed whether CR1 ligation influences the TLR driven immediate changes in the phenotype of B lymphocytes. To define this, isolated, resting, tonsillar B cells were treated with the synthetic TLR activators: R-837 (TLR7) or CpG ODN 2006 (TLR9). To engage concomitantly BCR and either of the TLRs, the F(ab')_2_ fragment of anti-human IgG/M/A was added to the cells simultaneously with one of the TLR-agonists. Crosslinking of CR1 was carried out by using its well-defined, multimeric ligand, C3b-like C3, prepared by heat aggregation of complement component C3 (3). Changes in the expression of the early activation marker CD69 and the costimulatory molecule CD40 were monitored after 48 h of culturing.

#### Clustering CR1 Does Not Affect the TLR7-Mediated Up-Regulation of CD69 and CD40, but Strongly Inhibits Their Expression Induced Upon Simultaneous Triggering via TLR7 and BCR

**Figure 1:**
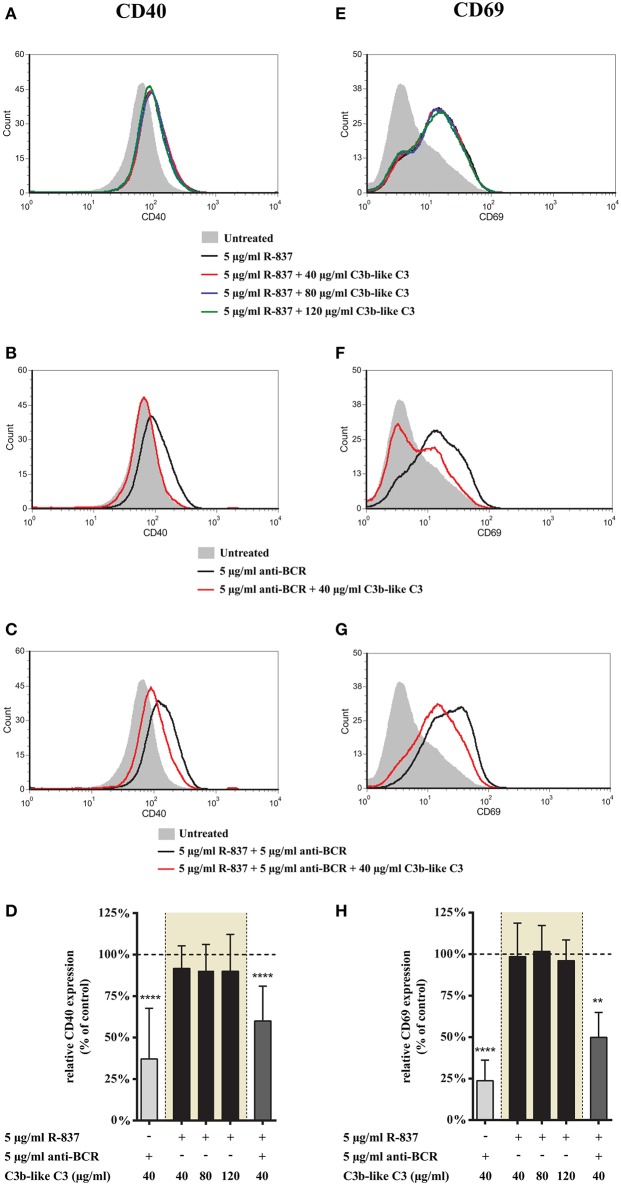
Effect of CR1 clustering on TLR7 and BCR induced upregulation of CD40 and CD69 on human B cells. B cells were activated for 48 h with 5 μg/ml R-837 or 5 μg/ml F(ab')_2_ anti-human IgG/M/A either separately or simultaneously, in the presence of different doses of C3b-like C3, or in the absence of the CR1 ligand (control, indicated as 100% in panels **(D,H)**. Changes in the expression of CD40 **(A–D)** and CD69 **(E–H)** were monitored by flow cytometry. Histograms of one representative measurement are shown in panels **(A–C,E–G)**. Relative mean effect of CR1 ligation on activation marker expression ± SD in samples stimulated with TLR7, BCR and both are calculated from six independent experiments **(D,H)**. **p < 0.01, ****p < 0.0001.

**Figure 2:**
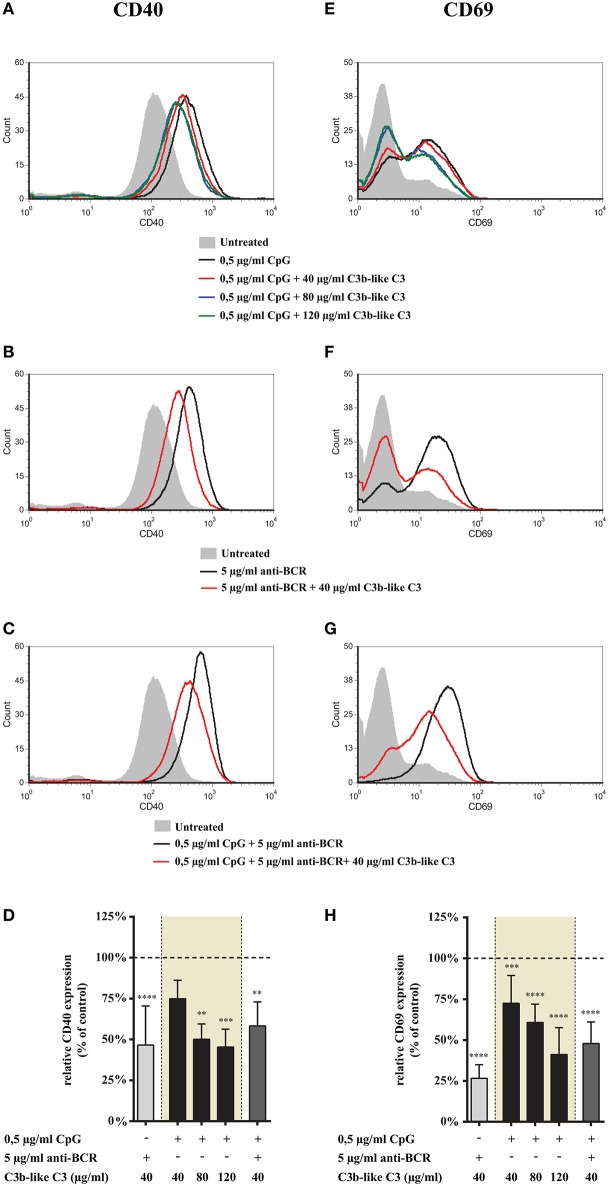
Effect of CR1 clustering on TLR9 and BCR induced upregulation of CD40 and CD69 on human B cells. B cells were activated for 48 h with 0.5 μg/ml CpG ODN 2006 or 5 μg/ml F(ab')_2_ anti-human IgG/M/A either separately or simultaneously, in the presence of different doses of C3b-like C3, or in the absence of the CR1 ligand (control, indicated as 100% in panels **(D,H)**. Changes in the expression of CD40 **(A–D)** and CD69 **(E–H)** were monitored by flow cytometry. Histograms of one representative measurement are shown in panels **(A–C,E–G)**. Relative mean effect of CR1 ligation on activation marker expression ± SD in samples stimulated with TLR9, BCR and both are calculated from at least three independent experiments **(D,H)**. **p < 0.01, ***p < 0.001, ****p < 0.0001.

First we studied how CR1 ligation affects the TLR7 induced marker expression of B cells. We found that neither CD40 nor CD69 expression changed upon crosslinking CR1, even when increasing doses of the complement-derived ligand were used (Figures 1A,D,E,H). Surprisingly however, upon concomitant engagement of TLR7 and BCR, even the lowest concentration of aggregated C3 caused a significant inhibition of both markers (Figures 1C,D,G,H), similarly to the case of single BCR stimulation (Figures 1B,D,F,H as well as Figures 2B,D,F,H).

These findings demonstrate that CR1 clustering does not affect the TLR7-induced phenotype change of human B cells, while it significantly inhibits the upregulation of CD40 and CD69 in B cells simultaneously activated by R-837 and anti-BCR.

#### CR1 Ligation Inhibits Up-Regulation of CD69 and CD40 on B Cells Activated via TLR9 Alone or Combined With BCR Ligation

Next, we examined the effect of CR1 ligation on the expression of activation markers induced by TLR9 alone and together with the BCR-stimulus. We found that engagement of CR1 significantly and dose-dependently inhibits the CpG induced up-regulation of CD40 (Figures 2A,D) as well as CD69 (Figures 2E,H). Importantly, the strongly elevated expression of these markers caused by the concomitant occupancy of TLR9 and BCR was also markedly diminshed already by the lowest concentration (40 μg/ml) of the natural ligand (Figures 2C,D,G,H).

These data show that CR1 clustering inhibits up-regulation of both CD40 and CD69 molecules on tonsillar B cells induced by TLR9 stimulation alone as well as in combination with BCR ligation.

### Influence of CR1 Ligation on Cytokine Production

Signaling via TLR7 and TLR9 directly activates B cells to secrete proinflammatory and immunoregulatory cytokines, out of which IL-6 is the most prominent (10, 15, 17). To this end we set out to study whether engagement of CR1 affects IL-6 production induced by TLR7 or TLR9 alone and in combination with BCR triggering. Cytokine production was measured from the supernatant of tonsillar B cells after stimulation for 48 h with the combination of various ligands, as shown in the figures.

#### Clustering CR1 Does Not Alter the TLR7 Triggered IL-6 Production of Tonsillar B Cells, but Potently Inhibits the Concomitant Activation via TLR7 and BCR

**Figure 3:**
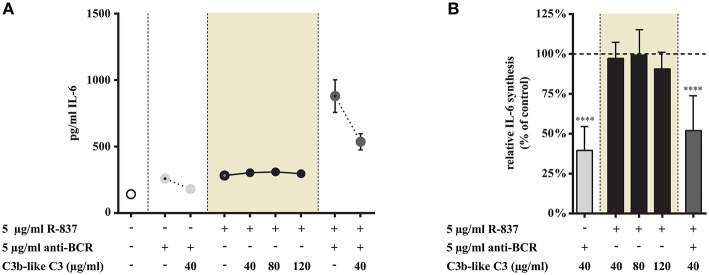
Influence of CR1 clustering on TLR7 and BCR induced IL-6 synthesis of B cells. B cells were activated for 48 h with 5 μg/ml R-837 or 5 μg/ml F(ab')_2_ anti-human IgG/M/A either separately or simultaneously, in the presence of different doses of C3b-like C3, or in the absence of the CR1 ligand (control, indicated as 100% in panel **(B)**. Supernatants were collected and examined for IL-6 production by ELISA. Data are expressed as mean concentration values (pg/ml) ± SEM of duplicate samples of a representative donor **(A)**. Relative mean effect of CR1 ligation on IL-6 synthesis ± SD in samples stimulated with TLR7, BCR and both, are calculated from seven independent experiments **(B)**. ****p < 0.0001.

We found that CR1 clustering did not affect IL-6 production induced by R-837, the TLR7 agonists, even if the ligand was used in increasing concentrations (Figure 3). At the same time however, both the BCR and BCR + TLR7 induced cytokine production was significantly reduced in the presence of C3b-like C3 (Figure 3).

Our data show that TLR7-triggered IL-6 production is not influenced by CR1 ligation, but the presence of the complement-derived ligand significantly and dose-dependently reduced the synergistic cytokine production of human B cells, induced by simultaneous activation by R-837 and anti-BCR.

#### CR1 Ligation Reduces the TLR9 Mediated IL-6 Production of Tonsillar B Cells Both in the Presence and Absence of Anti-BCR Stimulus

**Figure 4:**
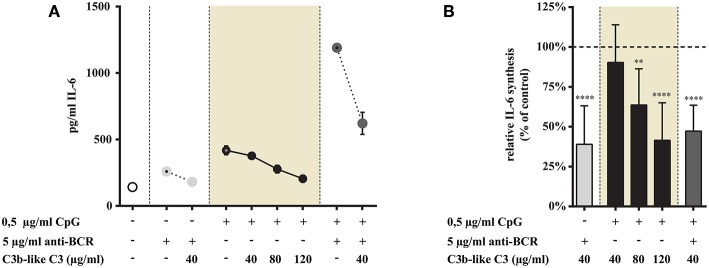
Influence of CR1 clustering on TLR9 and BCR induced IL-6 synthesis of B cells. B cells were activated for 48 h with 0.5 μg/ml CpG ODN or 5 μg/ml F(ab')_2_ anti-human IgG/M/A either separately or simultaneously, in the presence of different doses of C3b-like C3, or in the absence of the CR1 ligand (control, indicated as 100% in panel **(B)**. Supernatants were collected and examined for IL-6 production by ELISA. Data are expressed as mean concentration values (pg/ml) ± SEM of duplicate samples of a representative donor **(A)**. Relative mean effect of CR1 ligation on IL-6 synthesis ± SD in samples stimulated with TLR9, BCR and both, are calculated from seven independent experiments **(B)**. **p < 0.01, ****p < 0.0001.

As shown in Figure 4 we found that ligand binding to CR1 dose-dependently inhibits CpG induced IL-6 production (data in shaded background). We also demonstrate that the synergistically elevated IL-6 release—exerted by the simultaneous trigger via TLR9 and BCR—was significantly reduced by the engagement of CR1 (Figure 4).

These results show that CR1 clustering inhibits the enhanced cytokine production induced upon B-cell stimulation triggered by TLR9 alone as well as by TLR9 and BCR simultaneously.

### Impact of CR1 Clustering on Activation Induced Proliferation of Human B Cells

B cells are known to respond with enhanced proliferation both to the separate and the simultaneous trigger via the BCR and TLRs (24–26) while the engagement of CR1 has been shown to inhibit BCR-induced division of human B cells (3–5). Therefore, it was important to ascertain how the simultaneous presence of the two innate signals influences B cell growth. To this end we assessed the effect of CR1 ligation on TLR7 and TLR9 as well as on TLR7 + BCR and TLR9 + BCR induced proliferation, using different doses of the natural, multimeric ligand, C3b-like C3.

#### CR1 Occupancy Does Not Influence the TLR7 Induced Proliferation of Tonsillar B Cells, However It Significantly Inhibits the Simultaneous Trigger via BCR and TLR7

**Figure 5:**
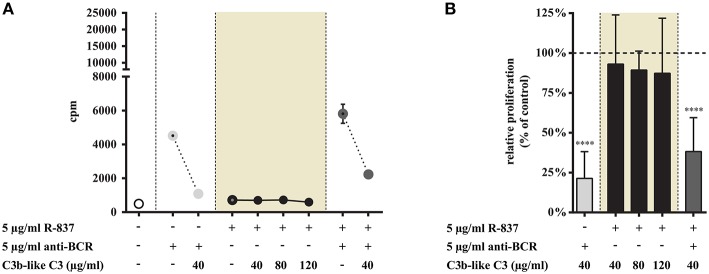
Impact of CR1 clustering on TLR7 and BCR induced proliferation of B cells. B cells were activated for 72 h with 5 μg/ml R-837 or 5 μg/ml F(ab')_2_ anti-human IgG/M/A either separately or simultaneously, in the presence of different doses of C3b-like C3, or in the absence of the CR1 ligand (control, indicated as 100% in panel **(B)**. Cells were harvested after pulsing with 1 μCi/well 3H-thymidine for the last 18 h of culture. Data are expressed as mean cpm (count per minutes) values ± SEM of triplicate samples of a representative donor **(A)**. Relative mean effect of CR1 ligation on proliferation ± SD in samples stimulated with TLR7, BCR and both, are calculated from six independent experiments **(B)**. ****p < 0.0001.

**Figure 6:**
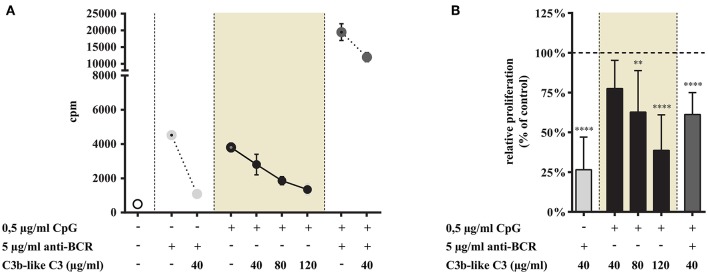
Impact of CR1 clustering on TLR9 and BCR induced proliferation of B cells. B cells were activated for 72 h with 0.5 μg/ml CpG ODN 2006 or 5 μg/ml F(ab')_2_ anti-human IgG/M/A either separately or simultaneously, in the presence of different doses of C3b-like C3, or in the absence of the CR1 ligand (control, indicated as 100% in panel **(B)**. Cells were harvested after pulsing with 1 μCi/well 3H-thymidine for the last 18 h of culture. Data are expressed as mean cpm (count per minutes) values ± SEM of triplicate samples of a representative donor **(A)**. Relative mean effect of CR1 ligation on proliferation ± SD in samples stimulated with TLR9, BCR and both, are calculated from six independent experiments **(B)**. **p < 0.01, ****p < 0.0001.

Stimulation of tonsillar B cells with R-837 resulted in a weak, double-fold proliferative response (Figure 5A) compared to the strong activation obtained when CpG was employed (Figure 6A), which is consistent with earlier results performed on blood derived B-cells (15, 16). This process was not influenced by C3b-like C3, even when the ligand was applied in much higher concentration, than the amount resulting in a significant inhibition of the BCR-trigger (Figure 5). Remarkable however, the proliferative effect exerted by the dual engagement of BCR and TLR7 was significantly reduced upon CR1 ligation (Figure 5).

These data are in line with the results obtained in our previous functional tests, demonstrating that in the case of TLR7 only the dual triggering (BCR + TLR7 stimulus) of B-cells could be inhibited by CR1 ligation. When we assessed the inhibitory capacity of CR1 on BCR-induced triggering of tonsillar B cells, we confirmed earlier results obtained with human blood-derived B cells (3–5) (Figures 5, 6).

#### Ligation of CR1 Inhibits TLR9 and TLR9 + BCR Induced Proliferation of Tonsillar B Cells

As shown in Figure 6 CR1 clustering significantly and dose-dependently inhibits the TLR9-driven proliferation of tonsillar B cells (data in shaded background). The combined stimulus of CpG and anti-BCR induced a synergistic proliferation of the cells, as known from literature (24–26) (Figure 6A). Importantly however, the presence of the natural ligand of CR1 significantly diminished even this very efficient proliferative response (Figure 6).

In contrast to TLR7, stimulation via TLR9 alone was strongly and dose-dependently inhibited by CR1 clustering. Our data demonstrate that the complement-derived ligand strongly diminishes even the synergistic proliferation induced by dual engagement of TLR9 and BCR.

### Impact of CR1 Crosslinking on Antibody Production

Stimulation of B cells with TLR7 and TLR9 agonists is known to result in B cell differentiation and antibody secretion with preferential IgM production (19). To assess whether antibody production induced by TLR stimulation alone as well as in conjunction with BCR engagement is affected by the complement-derived ligand, tonsillar B cells were cultured in the presence of different combinations of the activators—as detailed in the legend to figures. C3b-like C3 was added to the culture in different concentrations, and the number of IgM secreting cells was determined after 4 days by ELISpot assay.

#### Ligation of CR1 Does Not Influence IgM-Production Induced via TLR7, While It Suppresses Antibody Production Induced by the Concomitant Stimulus via TLR7 and BCR

**Figure 7:**
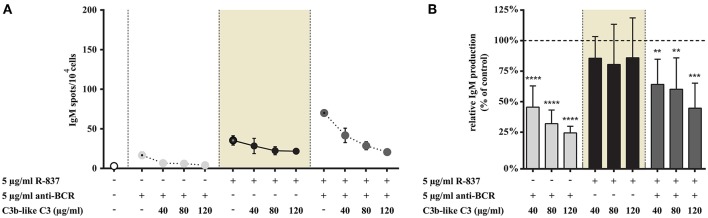
Impact of CR1 clustering on TLR7 and BCR induced IgM production of B cells. B cells were activated for 72 h with 5 μg/ml R-837 or 2 μg/ml F(ab')_2_ anti-human IgG/M/A either separately or simultaneously, in the presence of different doses of C3b-like C3, or in the absence of the CR1 ligand (control, indicated as 100% in panel **(B)**. Supplementary cytokines IL-2, IL-6, and IL-10 (50 ng/ml) were also added to the culture. The number of IgM-secreting cells was evaluated by ELISpot assay on anti-IgM-coated PVDF plates. Data are expressed as mean number of IgM spot values/10^4^ cells ± SEM of triplicate samples of a representative donor **(A)**. Relative mean effect of CR1 ligation on IgM production ± SD in samples stimulated with TLR7, BCR and both, are calculated from five independent experiments **(B)**. **p < 0.01, ***p < 0.001, ****p < 0.0001.

**Figure 8:**
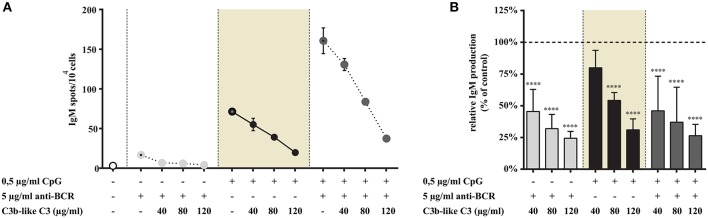
Impact of CR1 clustering on TLR9 and BCR induced IgM production of B cells. B cells were activated for 72 h with 0.5 μg/ml CpG ODN 2006 or 2 μg/ml F(ab')_2_ anti-human IgG/M/A either separately or simultaneously, in the presence of different doses of C3b-like C3, or in the absence of the CR1 ligand (control, indicated as 100% in panel **(B)**. Supplementary cytokines IL-2, IL-6, and IL-10 (50 ng/ml) were also added to the culture. The number of IgM-secreting cells was evaluated by ELISpot assay on anti-IgM-coated PVDF plates. Data are expressed as mean number of IgM spot values/10^4^ cells ± SEM of triplicate samples of a representative donor **(A)**. Relative mean effect of CR1 ligation on IgM production ± SD in samples stimulated with TLR9, BCR and both, are calculated from six independent experiments **(B)**. ****p < 0.0001.

In agreement with data published by others (15, 19) we also found that the TLR7 agonist was less effective than CpG in enhancing IgM production (Figures 7A, 8A), while stimulation of B cells via both TLR7 and BCR markedly enhanced the number of spots (Figure 7A). In the latter case CR1 ligation significantly and dose dependently decreased the frequency of IgM secreting cells—as illustrated in Figure 7. Nonetheless, the number of spots in solely R-837 treated samples did not change significantly (data shown in shaded background), even when C3b-like C3 was added to the culture in the highest concentration (120 μg/ml) (Figure 7).

Thus, similarly to our findings regarding various functions of human B cells, IgM-production induced by TLR7 alone was not influenced by the occupancy of CR1. The strongly elevated antibody production caused by the dual stimulus via TLR7 and BCR however, was significantly and dose-dependently inhibited in the presence of the complement-derived ligand.

#### CR1 Clustering Diminishes the Number of IgM Secreting B Cells Induced via TLR9 as Well as by the Simultaneous Engagement of TLR9 and BCR

Consistent with our previous results, stimulation with CpG or anti-BCR alone induced elevated IgM production, with synergism in the case of combined stimuli (Figure 8A). As shown in Figure 8, addition of the CR1 ligand to the cells inhibited spot formation strongly and dose-dependently in each case, including the highly elevated, synergistic effect of BCR, and TLR9 engagement.

In accordance with the results of our other functional assays, both the single TLR9 stimulus and the dual TLR9 + BCR engagement was significantly and dose-dependently inhibited by the complement C3-derived ligand.

## Discussion

In physiological conditions, when both complement and TLR activating microbial and damage products are present in the B cell environment, the interaction between the two innate sensory systems plays a crucial role in the regulation of B cell responses. Still, these two systems have been studied so far mainly as separate entities and their interplay is poorly understood. Previously we and others have shown that in human systems CR1 is a potent inhibitor of BCR-dependent B cell activation (3–7). Here we provide evidence that engagement of CR1 by its natural, complement-derived ligand concomitantly with either TLR7 or TLR9 stimulus significantly inhibits all major BCR-induced functions of human B cells—including activation marker expression, cytokine production, proliferation, and immunoglobulin production. Importantly, in the absence of the BCR stimulus, only TLR9 but not TLR7-induced activation is reduced, highlighting diverse mechanisms of the interplay between the inhibitory CR1 and these two TLRs in human B cells. Unfortunately the role of CR1 cannot be studied in mouse systems, since this inhibitory receptor is not expressed in these animals, as detailed in the Introduction.

Human naive B lymphocytes gain the ability to express TLRs—especially TLR9—only after BCR triggering, in contrast to the mouse system where the expression of TLRs in naive B cells is constitutive (27). Consequently in humans, TLR stimulation alone does not result in the activation of naive B cells, they require a combination of different signals for proliferation and differentiation (28). In contrast to this, human memory B cells can be directly activated by TLR agonists, due to their constitutive TLR expression (27). Therefore, we used B cells isolated from human tonsils for the experiments, which contain up to 50% memory cells (personal observation).

Consistent with data of other investigators (15, 16), we also found that CpG, the TLR9 agonist is a much more potent activator of B cells than R-837, which interacts with TLR7.

Results of mouse and human studies reveal that concomitant BCR and TLR7/TLR9 engagement results in synergistic B cell responses (24, 29–33)—including cytokine synthesis, antibody production, and class switch recombination -, due to the overlap between the BCR and TLR-mediated signaling pathways (29). This effect is further facilitated by the upregulation of TLR9 expression after BCR ligation (34, 35). In good agreement with these data, we also observed synergism as a consequence of combined TLR7+BCR and TLR9+BCR stimulation in most of the functional assays performed.

In addition to the proven inhibitory effect of CR1 on BCR-dependent functions (3–7) our present results reveal novel, so far undescribed mechanisms, and highlight an additional level of the regulation of human B cells. Data shown here unveil the importance of the crosstalk between the complement system and TLRs, which might take place under physilogical and pathological conditions.

B cells are known to respond to pathogen microbes not only through recall responses via the BCR, but through immediate TLR-dependent activation, as well. In the same time pathogens and altered self-structures may trigger the complement system, generating biologically active fragments leading to opsonization and inflammation. All these processes must be controlled to avoid pathological conditions. Our studies reveal that CR1—besides its well-known regulatory functions during the complement activation cascade—plays a pivotal role by decreasing early polyclonal activation of B cells, via binding of C3b-opsonized particles or complexes.

The size of the C3b-like C3 we used was found to be ~2–3-fold bigger than the non-aggregated molecule by electron microscopic analysis (data not shown). Thus, it can be considered as a multimeric, natural ligand of CR1, which clusters the receptors, similarly to C3b-opsonized antigens appearing *in vivo*. We have thoroughly characterized the properties of C3b-like C3 in our earlier published paper (3). We clearly proved by ELISA that it exposes C3b—but not C3d—epitopes, and demonstrated by FACS analysis that it binds specifically to CR1 only—and not to CR2. Thus, the possible effect of CR2 occupancy by C3b-like C3 can be excluded. We can also exclude the possible involvement of other C3-derived fragments which might be generated locally—such as iC3b and C3d—since our earlier results demonstrated that ligation of CR1 induces immediate changes in the BCR-induced signaling in human B-cells. Namely, addition of C3b-like C3 inhibits promptly both the BCR-induced Ca^2+^-influx (3), and the phosphorylation of Syk, Erk, and JNK (5).

Since most complement proteins that are present in serum do not reach all sites in the body, complement components synthesized in various tissues might be involved in the described processes. The level of local concentration depends on the activation state of different cell types known to produce complement factors—including macrophages, dendritic cells, epithelial cells. Locally produced complement proteins have already been shown to play a role in the initiation and/or regulation of the immune response [reviewed in (36, 37)].

CR1 has the capacity to down-regulate B lymphocyte functions even when the cells are triggered via TLR9 only, which is important in the BCR-independent regulation of memory B cell functions. However, the effect of clustering CR1 on solely TLR7 or TLR9 induced activation was markedly different. While engagement of CR1 inhibited the CpG-induced activation significantly, it did not affect the TLR7 mediated functions, even when C3b-like C3 was employed in the highest concentration (120 μg/ml). Furthermore, it is equally important to emphasize, that when either TLR7 or TLR9 are engaged simultaneously with the BCR, the strong inhibitory effect of the complement-derived ligand prevails, demonstrating that the BCR-dependent powerful and synergistic activation is also efficiantly downregulated by CR1. However, in the case of combined TLR7 and BCR activation CR1 ligation most probably affects only the BCR-dependent component of the response. This is supported by our marker expression studies showing that CD40 and CD69 expression was similar in dual TLR7+BCR engaged samples after CR1 ligation and single TLR7 triggered samples.

We suppose that CR1 exerts its inhibitory effect by interfering with the signaling pathways involved in both BCR and TLR9 induced activation of human B cells, but it is independent from the TLR7-mediated intracellular events. Several signaling molecules downstream of the BCR operate in concert with the TLR pathways to positively modulate B cell responses [reviewed in Frontiers in Immunology, in 2017 by Suthers (29)]. Out of these the spleen tyrosine kinase, Syk has long been known as a key molecule in the activation of B lymphocytes (38). Although CR1-signaling has not been extensively studied so far, our previous results show that Syk is involved in the CR1 mediated inhibition, since the BCR-induced phosphorylation of this tyrosine kinase is reduced by CR1 ligation (5). Furthermore, it has also been proven that the TLR9-mediated activation of B cells is diminished in the presence of Syk inhibitors (14, 39), due to the involvement of Syk in the delivery of cell surface TLR9-bound CpG into TLR9-containing endolysosomes (14). In contrast to TLR9, Syk has not been shown to be involved in TLR7 signaling, which might be explained by the lack of TLR7 expression on the cell surface.

It has been reported that synergistic BCR-TLR7/9 activation by nucleic acid—protein complexes are involved in the promotion of autoreactive B cells via the induction of increased proliferation and autoantibody production (40–43). Furthermore, full activation of autoreactive rheumatoid factor B cells requires these dual signals as well (30, 44). It has also been shown that combined stimulation via the BCR and TLR7 leads to the attenuation of peripheral B cell tolerance (45). Surprisingly however, dual engagement of BCR and TLR9 can lead to opposing functional outcome. In autoreactive B cells induction of tolerance through activation-induced cytidine deaminase (AID) expression was observed by Kuraoka et al. as a consequence of synergistic BCR and TLR9 signals (46). It is important to note, that the CR1-mediated inhibition concerns the entire memory pool, since TLR7/9 as well as CR1 are constitutively expressed on human memory B-cells. This might be particularly important in the control of autoimmune diseases.

Overall, we conclude that CR1 (CD35) has an important role in maintaining the balance between normal and pathological B cell activation. We assume that this complement receptor might be considered as a potential therapeutical target in systemic lupus erythematosus (SLE) and rheumatoid arthritis (RA), to decrease activation and antibody production by autoreactive B cells. Succesful elimination of pathological B lymphocytes by targeting CR1 has already been demonstrated in a humanized SCID mouse model of SLE (7).

## Data Availability

The datasets generated for this study are available on request to the corresponding author.

## Ethics Statement

Tonsils from children undergoing routine tonsillectomy were obtained from the Saint Istvan and Saint Laszlo Hospital in Budapest. This study was carried out in accordance with the Helsinki Declaration, and the study was approved by the Ethics Committee of the Medical Research Council in Hungary (TUKEB), 52088/2015/EKU.

## Author Contributions

BM-V and AE designed the study, interpreted results, and prepared the manuscript. BM-V, KN, and LF performed experiments. AE supervised research.

### Conflict of Interest Statement

The authors declare that the research was conducted in the absence of any commercial or financial relationships that could be construed as a potential conflict of interest.
